# Internet-Based Recruitment and Retention of Young Adults With Type 1 Diabetes: Cross-Sectional Study

**DOI:** 10.2196/46415

**Published:** 2023-08-22

**Authors:** Stephanie Griggs, Garrett I Ash, Grant Pignatiello, AnnMarie Papik, Johnathan Huynh, Mary Leuchtag, Ronald L Hickman Jr

**Affiliations:** 1 Case Western Reserve University Frances Payne Bolton School of Nursing Cleveland, OH United States; 2 Yale School of Medicine New Haven, CT United States

**Keywords:** type 1 diabetes, internet-based recruitment, young adult, diabetes, diabetic, type 1, recruit, research platform, T1D, social media, research subject, research participant, study participant

## Abstract

**Background:**

Multiple research strategies are required to recruit and engage a representative cohort of young adults in diabetes research. In this report, we describe an approach for internet-based recruitment for a repeated-measures descriptive study.

**Objective:**

The objective of this cross-sectional study was to determine whether internet-based recruitment through multiple social media platforms, a clinical research platform, and cooperation with community partnerships—College Diabetes Network and Beyond Type 1—would serve as an effective way to recruit a representative sample of young adults aged 18-25 years with type 1 diabetes (T1D).

**Methods:**

We conducted a repeated-measures descriptive study. We captured enrollment rates and participant characteristics acquired from each social media platform through survey data and Facebook analytics. This study was advertised via paid postings across a combination of different social media platforms (eg, Facebook, Instagram, Twitter, and Reddit). We used quarterly application postings, quarterly newsletters, and participation in the ResearchMatch registry to identify potentially eligible participants from February 3, 2021, to June 6, 2022.

**Results:**

ResearchMatch proved to be the most cost-effective strategy overall, yielding the highest gender and racial diversity compared to other internet platforms (eg, Facebook, Instagram, Twitter, and Reddit), application postings (eg, Beyond Type 1), and newsletters (eg, College Diabetes Network and a local area college). However, we propose that the combination of these approaches yielded a larger, more diverse sample compared to any individual strategy. Our recruitment cost was US $16.69 per eligible participant, with a 1.27% conversion rate and a 30% eligibility rate.

**Conclusions:**

Recruiting young adults with T1D across multiple internet-based platforms was an effective strategy to yield a moderately diverse sample. Leveraging various recruitment strategies is necessary to produce a representative sample of young adults with T1D. As the internet becomes a larger forum for study recruitment, participants from underrepresented backgrounds may continue engaging in research through advertisements on the internet and other internet-based recruitment platforms.

## Introduction

Young adults with type 1 diabetes (T1D) achieve glycemic targets at the lowest rates compared to other age groups [1]. However, there is a limited research focus on this specific age group [2,3]. Innovative, inclusive, and accessible approaches are required to address the individual circumstances preventing young adults with T1D from achieving optimal diabetes and overall health [4,5]. Further, the transition from pediatric to adult care is a difficult time to enroll individuals in research who are often hard to reach due to psychosocial or logistical constraints [6-8]. Traditionally, clinical researchers leverage health care system relationships to engage with this high-risk population. However, clinic recruitment elicits a selection bias and may yield findings not representative of social or economic disadvantages.

Social media is a promising recruitment avenue for reaching young adults, as a vast majority use social media and seek internet-based health information (88% and 94%, respectively) [9]. Additionally, there are several T1D peer and role model support groups hosted on social media [10,11]. Recruiting young adults with T1D from social media group advertisements results in selection bias, a trend consistent with previous studies [12,13], as not all young adults with T1D engage with these groups. Other web-based platforms, like Reddit, Twitter, and designated recruitment services (eg, ResearchMatch), are alternative modes for recruiting these underrepresented research participants. To our knowledge, a combination of these other platforms with traditional internet-based recruitment (eg, predominantly Facebook) of young adults with T1D has not been previously used. Therefore, in this repeated-measures descriptive study, we aimed to determine whether internet-based recruitment through multiple social media platforms (eg, Facebook, Instagram, Twitter, and Reddit), ResearchMatch (a clinical research platform), and cooperation with community partnerships—College Diabetes Network (CDN) and Beyond Type 1 would serve as an effective way to recruit a representative sample of young adults aged 18-25 years with T1D.

## Methods

### Community-Engaged Research or Partnership Building

CDN and Beyond Type 1 were contacted for cooperation to assist in recruiting young adults with T1D. These groups are nonprofit organizations designed to connect young adults with T1D and are focused on education, advocacy, peer support, and providing resources. The groups were contacted to determine their target population, the extent of their outreach, and their information distribution methods. CDN uses a newsletter and reaches over 160 universities across the United States. Beyond Type 1 reaches a broad college and working adult audience across the United States using blogs and social media.

### Study Procedures and Design

The intent of this cross-sectional study was to determine whether internet-based recruitment through multiple social media platforms, a clinical research platform, and cooperation with CDN and Beyond Type 1 would serve as an effective way to recruit a representative sample of young adults with T1D. This study was advertised via paid postings across a combination of different social media points (eg, Facebook, Instagram, Twitter, and Reddit). These postings were monitored weekly by research staff. We used quarterly application postings (via Beyond Type 1), quarterly newsletters (via CDN and a local area college), and participation in the ResearchMatch registry to identify potentially eligible participants from February 3, 2021, to June 6, 2022. ResearchMatch, a national health volunteer registry, was created by several academic institutions and supported by the US National Institutes of Health as part of the Clinical and Translational Science Award Program. ResearchMatch has a large population of volunteers who have consented to be contacted by researchers about health studies for which they may be eligible. Volunteers who sign up for ResearchMatch complete a baseline survey with demographic details on age, contact information, and optional health questions. A short description of the study, inclusion criteria, and incentives were used within the postings on social media and within ResearchMatch with a direct link to the screening survey.

We used a repeated-measures design to follow participants over 7 days. Baseline survey data were used to determine participant demographics, study advertisement location, medical history, and sleep health dimensions. Raw time-series data from each participant’s continuous glucose monitor (CGM) were obtained from participant-uploaded raw data (eg, .csv or .xlx file exports) or by downloading data from a participant-provided share-code (Clarity for Dexcom users only). Those using other devices uploaded their raw data exports directly to REDCap (Research Electronic Data Capture; eg, FreeStyle Libre and Medtronic). CGM data were collected to describe the study sample.

### Ethics Approval

The Case Western Reserve University Institutional Review Board approved the study procedures (STUDY20201829) and ensured that ethical principles were applied to research activities. All participants signed informed consent to participate in this study. Study staff were trained in confidentiality and data security procedures. All data were deidentified, coded with a unique identifier, and securely stored separately from any personally identifiable information. Access to data storage was restricted to authorized study personnel.

### Eligibility Criteria and Screening Procedure

**Figure 1:**
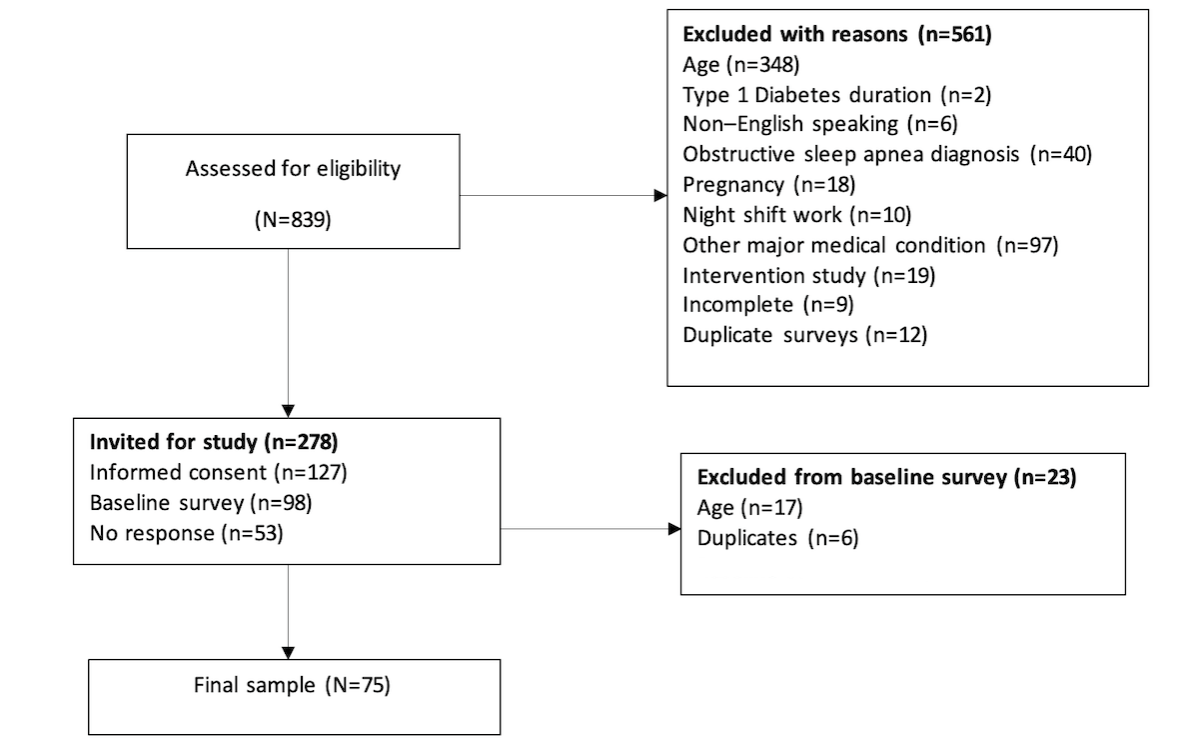
Study screening and enrollment flow diagram.

We used a 9-item screening survey to identify eligible participants. This brief survey screened for the inclusion criteria, which are the following: (1) between the ages of 18-25 years, (2) diagnosed with T1D for at least 6 months, (3) without a previous obstructive sleep apnea diagnosis or other major medical or severe psychiatric conditions, (4) not participating in intervention studies, (5) able to read or speak English, (6) not currently pregnant, and (7) not working night shifts. This brief internet-based questionnaire assessed the eligibility criteria, and participants who were screened eligible provided written informed consent before study enrollment. Participants were compensated with two US $10 electronic incentives for completion of internet-based data collection: one for the 30-minute baseline survey and the second for the 3-5 minute twice-daily diaries. Participants were also provided with a national resource guide, including options for emergencies, mental health treatment, domestic violence, abuse, sexual assault, and diabetes resources. We present the flow of the study screening and enrollment in Figure 1.

### Procedures to Ensure Data Validity

We used several strategies to promote data validity and legitimate participation. The REDCap link was protected with reCAPTCHA, an advanced risk analysis engine with adaptive capability to block automated spam software. Research staff assessed the screening survey for duplicate emails, email patterns (eg, 2 names followed by “ABC” or “123”), or a pattern of implausible values. The baseline survey was monitored to prevent further progression through the repeated-measures study when invalid or ineligible cases were identified. We reported ineligible cases to the institutional review board and were instructed to track these as screen failures.

### Statistical Analysis

Analyses were performed using SPSS software (version 28; IBM Corp). We used descriptive statistics to describe the overall sample and to determine the acceptability of the research study. The 4 groups were compared using a one-way ANOVA. Statistical significance was set at *P*<.05. The 4 groups were comprised of Facebook, CDN, ResearchMatch, and others (ie, Twitter, Reddit, and Beyond Type 1). We collapsed Twitter, Reddit, and Beyond Type 1 due to the small cell count for each group (<5). The Glyculator 3.0, a glycemic variability calculation tool, was used to compute CGM-based glucose metrics (eg, mean, time in range, and glucose management indicator in mg/dL) based on international consensus guidelines [14].

Impressions were determined by the number of times that the ad was fetched using Facebook analytics. The cost per click was determined by the cost of advertising or the number of times the advertisement was clicked in US dollars. The conversion rate was the percentage of participants who clicked on the advertisement and enrolled in the study. Further, this conversion rate reflects the specificity of ad campaigns targeting eligibility. The cost per participant was determined by the cost of advertising divided by the eligible recruited participants.

## Results

### Screening for Inclusion

Of 839 potentially eligible participants (824 selected through the screening survey initiated by different social media postings rotated weekly as well as quarterly CDN updates for advertising and 15 selected through ResearchMatch registry presence), 278 (33.1%) were eligible and invited to participate with an informed consent link (Figure 1). Participants were ineligible based on age (n=348, 41.5%), other major medical conditions (n=97, 11.6%), obstructive sleep apnea (n=40, 4.8%), and other reasons (n=76, 9.1%). Other reasons included T1D duration <6 months (n=2, 0.2%), non–English speaking (n=6, 0.7%), pregnancy (n=18, 2.1%), night shift work (n=10, 1.2%), intervention study participation (n=19, 2.3%), incomplete survey (n=9, 1.1%), and duplicates (n=12, 1.4%).

Close to half (n=127, 45.6%) of the eligible participants completed the informed consent, and 98 (35.3%) completed the internet-based baseline survey. We further excluded 23 due to age (n=17, 17.3%) and fraudulent or duplicate data (n=6, 6.1%). The twice-daily diaries were initiated by 55 (73.3%) and completed by 38 (≥ 60% completion) participants; 43 (57.3%) provided CGM data at baseline and 33 (44%) at the end of the 1-week study.

### Sample

The mean age of our final sample (n=75) ranged from 21.0 (SD 2.1) years to 21.9 (SD 2.3) years. Male sex assigned at birth accounted for 33% (n=25), and gender minority represented 9% (n=7) of the sample. Facebook and ResearchMatch yielded a higher percentage of male sex assigned at birth per platform (n=8, 50% and n=3, 23%, respectively) compared to CDN and the other sources (n=5, 18% and n=2, 12%, respectively; *P*=.03). ResearchMatch and platforms other than Facebook and CDN yielded greater gender, racial, and ethnic diversity. We were unable to evaluate differences in individuals identifying as a gender minority due to the small cell count.

There were differences for sex assigned at birth across platforms, with the most even distribution for male sex via Facebook (n=8, 50%) and a lower representation of the male sex via ResearchMatch (n=3, 23%), the CDN newsletter (n=5, 18%), and other platforms (n=2, 12%; *P*=.03). Differences were not significant across recruitment platforms for other demographic, diabetes, or sleep characteristics (*P*>.05). Participant demographics and clinical profiles are presented in Tables 1 and 2.

We present the recruitment source in Table 3. Advertisements were seen across social media platforms (eg, Facebook, Twitter, and Reddit) and CDN. From February 23, 2021, to April 29, 2022, our Facebook advertisement received 146,449 impressions with 1257 clicks on the advertisements. We spent approximately US $1 on average, with a 1.27% conversion rate, 30% eligibility rate, and a cost of US $16.69 to recruit each eligible participant. Facebook Analytics are presented in Table 4. The other platforms did not have a cost associated for advertising.

**Table 1:** Participant demographic and clinical characteristics (N=75).

Characteristics			Recruitment source, n (%)									
			Facebook		CDN^a^ newsletter		ResearchMatch		Other^b^		*P* value	
Total, n (%)			16 (21)		28 (37)		13 (17)		18 (24)		—^c^	
Age (years), mean (SD)			21.4 (1.9)		21.0 (2)		22.0 (1.6)		21.8 (2.5)		.58	
Sex assigned at birth (male), n (%)			8 (50)		5 (18)		3 (23)		2 (12)		.03	
**Gender, n (%)**												—
	Woman or female	8 (50)		21 (75)		8 (62)		12 (71)				
	Man or male	8 (50)		5 (18)		3 (23)		2 (12)				
	Genderqueer	0 (0)		1 (4)		1 (8)		0 (0)				
	Nonbinary	0 (0)		1 (4)		1 (8)		2 (12)				
	Trans man	0 (0)		0 (0)		0 (0)		1 (6)				
BMI (kg/m^2^), mean (SD)			25.4 (5.3)		24.2 (4.3)		25.1 (5.5)		24.3 (4.0)		.68	
**Race, n (%)**												
	Asian	1 (6)		0 (0)		2 (15)		1 (6)		.22		
	Black or African American	0 (0)		1 (3.6)		1 (8)		3 (18)				
	Multirace	1 (6)		0 (0)		0 (0)		0 (0)				
	White	14 (88)		27 (96)		10 (77)		13 (77)				
Ethnicity (Hispanic or Latino), n (%)			0 (0)		1 (4)		1 (8)		3 (18)		.08	

^a^CDN: College Diabetes Network.

^b^Other recruitment sources: Twitter, Reddit, and Beyond Type 1.

^c^Not applicable.

**Table 2:** Diabetes profile (N=75).

Characteristics		Recruitment source					
		Facebook	CDN^a^ newsletter	ResearchMatch	Other^b^	*P* value	
Type 1 diabetes duration (years), mean (SD)		7.7 (7)	10.1 (5)	11.4 (4.9)	8.2 (5.9)	.70	
A_1c_ (%), mean (SD)		7.1 (1.1)	6.7 (1.1)	6.6 (1.0)	7.1 (1.0)	.78	
Insulin pump (yes), n (%)		9 (56)	23 (82)	8 (62)	7 (41)	.07	
**Continuous glucose monitor, mean (SD)**							
	GMI^c^ (%)	7.6 (0.66)	6.9 (0.53)	7.0 (0.60)	7.1 (0.83)	.06	
	Glucose (mg/dL)	180.1 (27.5)	148.8 (21.9)	154.3 (25.2)	160.4 (34.8)	.06	
	Time in range (% 70-180mg/dL)	57.2 (14.8)	73.9 (13)	71.2 (19.8)	66.1 (23.4)	.09	
**Brand, n (%)**							.87
	Dexcom G6	9 (56)	24 (86)	10 (77)	8 (47)		
	Dexcom G5	1 (0)	0 (0)	0 (0)	2 (12)		
	Guardian	3 (19)	1 (4)	1 (8)	1 (6)		
	Enlite	1 (6)	0 (0)	0 (0)	1 (6)		
	FreeStyle Libre	0	2 (7)	1 (8)	2 (12)		
	None	2 (13)	1 (4)	1 (8)	3 (18)		

^a^CDN: College Diabetes Network.

^b^Other recruitment Source: Twitter, Reddit, and Beyond Type 1.

^c^GMI: glucose management indicator, derived from continuous glucose monitor data.

**Table 3:** Recruitment source (N=75).

Recruitment source	Values, n (%)
College Diabetes Network	28 (37)
Facebook	16 (21)
ResearchMatch	13 (17)
Other^a^	18 (24)

^a^Other recruitment sources included Twitter (n=4), Reddit (n=6), and Beyond Type 1 app or website (n=8).

**Table 4:** Social media analytics.

Analytics	Facebook (February 23, 2021, to March 29, 2022)
Impressions, n	146,449
Clicks, n	1257
Cost per click (US $)	1
Conversion rate (N=1257), n (%)	16 (1.27)
Eligibility rate (N=825), n (%)	246 (30)
Cost per participant (US $)	16.69
Cost per all 75 participants (US $)	1251.67

## Discussion

### Principal Findings

The purpose of this study was to determine whether internet-based recruitment through multiple social media platforms, a clinical research platform, and cooperation with CDN and Beyond Type 1 would serve as an effective way to recruit a representative sample of young adults with T1D. We focused on social media outreach of young adults aged 18-25 years with T1D and found that internet-based recruitment was a feasible and cost-effective method to recruit this hard-to-reach population. Our findings build upon previous studies of adults with T1D [12,13]. For instance, in a study of adults across a larger age range, internet-based methods were found to be more cost-effective and efficient compared to traditional methods [12]. Though all avenues of recruitment yielded participants eligible for the study, it was found that groups of individuals with certain characteristics, such as optimal glycemic targets, were more routinely recruited through specific sources, which suggests that outreach through multiple sources is needed to allow for representativeness in study sampling. Significant sociodemographic and health characteristic differences between individuals recruited from CDN and Beyond Type 1 were reported in another internet-based study of young adults with T1D [13].

Essential elements, as defined by cost per ad click, conversion rate, eligibility, and cost per participant, were evaluated as suggested by Whitaker and colleagues [15] in a comprehensive review of 35 internet-based studies. Compared to the review of similar studies by Whitaker and colleagues [15], we had comparable impressions (146,449 in our study vs 12,900,000) [15] relevant to the 825 participants who filled out the screening form linked to the advertisement; our ad ran longer than the mean duration in similar studies (14 months in our study compared to a mean of 5.13 months [15]), had a lower conversion rate (1.27% in our study vs 7%), lower eligibility rate (30% in our study vs 65%), higher cost per ad click (US $1 vs a mean of US $0.57), and a lower cost per participant (US $16.69 in our study vs US $19.77). The lower conversion rate indicates that prolonged advertisements may increase impressions but may not increase the number of people screened [15].

ResearchMatch yielded a higher percentage of male sex assigned at birth and the highest percentage of individuals identifying as racially, ethnically, or gender diverse in our study. These are key characteristics to assess when determining whether sampling is effective, especially in this hard-to-reach population. Multiple participants reported nonbinary gender identities when filling out their surveys, indicating that recruiting through social media may be an effective approach to recruiting a gender-diverse sample. Our sample had a more even distribution of male and female sexes at birth (n=49, 65% female) in comparison to a similar study (80.4% female) [13]; we also achieved a comparable representation of other races when compared to the national T1D clinic registry [1].

It is important to interpret the study findings within the context of the limitations and strengths of the web-based recruitment methods used. First, our findings are preliminary, given the small sample size, and may not generalize to other populations and settings. Second, we were unable to determine how many individuals saw the social media ads or how many newsletters or messages on platforms were effectively delivered and read. Third, though we excluded surveys with implausible values, suspicious patterns, or duplicates, individuals may have used another email, and this might have created a duplicate record. Lastly, recruitment depended on who was using social media at the time to view the advertisement.

The efficiency of web-based recruitment strategies to enroll participants in studies has been documented in previous studies [12,15]. However, in our study, we used social media in addition to traditional internet-based recruitment to find trends among participants recruited from various sources to purposefully increase diversity within our sample. We purposefully separated sex assigned at birth from gender identity; however, in a majority of previous studies, sex and gender are combined with only binary choices. Though gender and sex are oftentimes used interchangeably in the literature, it is important to note that both aspects contribute to an individual’s identity. Sex at birth refers to hormones as well as primary and secondary reproductive organs. Sex at birth and gender identity correspond for people who identify as cisgender but conflict for those identifying as transgender. Additionally, the binary sex option does not appropriately describe genderqueer individuals—who do not identify with any specific gender norms—nor does it describe nonbinary individuals—who identify neither as male nor as female [16]. Thus, the generalizability in this study is limited; this work will contribute to the limited literature distinguishing sex and gender identity in young adults with T1D.

### Conclusions

Recruitment of young adults aged 18-25 years with T1D has traditionally occurred through in-person clinician visits; however, internet-based recruitment is becoming increasingly popular, as young adults engage in social media platforms, ResearchMatch, and other internet-based community outreach forums. Based on our findings, we suggest that internet-based recruitment yields moderately diverse samples that better represent target study populations. As the internet becomes a larger forum for study recruitment, participants from underrepresented backgrounds may continue engaging in research through advertisements on the internet and other web-based recruitment platforms.

## Abbreviations

CDN: College Diabetes Network
CGM: continuous glucose monitor
REDCap: Research Electronic Data Capture
T1D: type 1 diabetes

## Data Availability

The data sets generated and analyzed during this study are available from the corresponding author upon reasonable request.
